# The implementation of a community-centered first aid education program for older adults—community health workers perceived barriers

**DOI:** 10.1186/s12913-023-09142-y

**Published:** 2023-02-08

**Authors:** Guo Yin, Linghui Chen, Yuanrong Wu, Fei Zhao, Qian Zhu, Siting Lin

**Affiliations:** 1 School of Nursing, Sanquan College of Xinxiang Medical University, Xinxiang City, Henan China; 2 Florence Nightingale Faculty of Nursing, Midwifery & Palliative Care, King’s College, London, United Kingdom; 3 School of Nursing and Health, Nanfang College, Guangzhou, Guangzhou city, Guangdong China; 4 Nursing Department, Second People’s Hospital of Qujing City, Qujing City, Yunnan China

**Keywords:** First aid education, Older adults, Community, Community health workers

## Abstract

**Background:**

Older adults are a high-risk group for accidental injuries, and strengthening training in first aid for older adults can improve their first aid capabilities and minimize their post-accident mortality. Community health workers are the greatest option to equip older adults with first aid instruction and training. However, the development of first aid education for the public by community health workers fails to take into account the elderly population. In view of this, this study aims to explore the barriers and challenges of first aid training for older adults from the perspective of community health workers and to provide a basis for better first-aid training for older adults in the community.

**Methods:**

This study adopted a qualitative research design. A total of 18 community health workers were recruited from two community health service centers in Qujing and one community health service center in Guangzhou from May to July 2022 to participate in the study. Participants were interviewed face-to-face using semi-structured in-depth interviews. The interview data were analyzed using Krippendorff's thematic clustering technique.

**Results:**

The results of the study identified community health workers' perceived challenges and barriers to providing first aid training to older adults in the community as older adults-level barriers, community health workers-level barriers, management systems–level barriers, and society-level barriers.

**Conclusions:**

Community health workers are highly aware of multiple barriers and challenges in providing first aid training to community-based elderly populations. In particular, lack of professional training, heavy workloads, and limited resources and financial support. Therefore, supportive training, policies, and government funding are crucial for community health workers to conduct first aid training for older adults.

## Introduction

With the prolongation of life expectancy, the aging degree of China's population continues to increase, as does the number of older adults [1]. The physical and mental changes associated with aging and frailty increase the risk of unintentional injuries in older adults [2]. Unintentional injuries and accidents are the major causes of death among older adults, including traffic accidents, cuts, choking, burns, and falls [3]. Falls are a common type of injury among older adults [4]. Moreover, most cardiac arrest victims are also older adults due to their age group's high prevalence of cardiovascular disease [5]. Due to the vulnerability of older adults to unintentional injuries also leads to an increase in the rate of emergency department care for older adults, which also increases costs to the healthcare system and the economic burden on society [6].

On the other hand, the number of empty nesters is growing fast due to decreased fertility rates, young people living independently, and population mobility [7]. By 2030, it is anticipated that empty nesters aged 60 and above will account for 90% of the elderly population in China [8]. "Empty nesting" refers to older adults living alone or with their spouses without children [9]. The lack of care and support from children, relatives, and friends for "empty nesters" exposes them to a higher risk of unintentional injuries. Multiple studies have also demonstrated that lack of family supports increases the probability of mortality in older persons following acute health crises [10, 11]. Therefore, it is crucial for older adults to have first aid skills.

First aid (FA) refers to the skills anyone can use to assess and intervene in injury without medical equipment [12]. Although FA measures cannot prevent injuries such as burns, stroke, or cardiac arrest, they can mitigate the consequences of these unintentional injuries and even save lives in some cases [13]. In the case of scalding, a 20-min cold-water rinse can halt the burn progress and improve the burn results [14]. Additionally, bystander provision of effective cardiopulmonary resuscitation (CPR) immediately after cardiac arrest can increase a person's chances of survival by 30% [15]. FA training aims to educate the public on these life-saving techniques to help prevent the casualty from deteriorating and improve prognosis [16]. Typically, Older adults are regarded as a group to be rescued, but they actually can play an essential role in self-rescue and rescuing others. For example, sudden cardiac death usually occurs in older adults around 60, and the bystanders most likely to initiate CPR are older adults, mostly the patient's spouse [17]. Phung et al. (2017) further stated that anyone could perform self-rescue and rescue others through standardized training in FA knowledge and skills [18]. Therefore, enhancing the training of FA skills for older adults in the community can improve their FA capabilities and reduce their post-accident mortality.

Community health workers (CHWs) undertake various roles, including health education, consultation, and coordination for residents in the community, and can disseminate FA knowledge and skills to older adults [19]. Therefore, CHWs are the best option to provide first aid education and training for older adults in the community. A Community Health Work (CHW) is broadly defined as a public health worker who resides in the community they serve and is chosen by community members [20]. However, in China, CHWs are usually medically trained physicians or nurses who work in the community. In fact, there is no common name that applies to all CHWs in China [21, 22], and CHWs have different names for their assigned tasks, such as community health volunteers, barefoot doctors, and village health workers [23]. To avoid confusion, the term "CHWs" will be used throughout this study to refer to "medically trained community health workers."

Although community health service workers are critical to improving the knowledge and skills of first aid for older adults. However, accidental injury of older adults is a relatively neglected health issue, and the FA education provided to the public by CHWs fails to account for the elderly population [24, 25]. In light of this, this study conducted in-depth interviews with CHWs to explore their perspectives on FA training for the elderly population. Identify CHWs perceived barriers and challenges in conducting FA training for older adults and provide a foundation for improved FA training for older adults in the community.

## Method

### Study Design

This study used a qualitative research design to explore participants' perspectives to understand phenomena rather than generalize findings [26]. This study adhered to the Comprehensive Criteria for Reporting Qualitative Research (COREQ) guidelines [27]. In addition, this qualitative study strictly followed the four criteria of qualitative research rigor in terms of method: credibility, transferability, dependability, and confirmability [28].

Participants were recruited from two community health service centers in Qujing, China, and one community health service center in Guangzhou, China, from May to July 2022. The researchers first contacted the directors of community health centers by telephone. Directors are responsible for relaying information and details of the study to their community health workers. CHWs interested in participating in the study contacted the researcher by phone. To obtain more accurate and comprehensive information, CHWs who have worked in community health centers for more than six months were eligible to participate in the study. In addition, CHWs from different locations, settings (rural, urban), education, age, and working years were selected to ensure that the selected participants were representative of the various perspectives of community health workers in different settings to ensure the transferability of the study findings [28]. Researchers scheduled interview times and venues with eligible CHWs.

To ensure the transferability of the study results, the sample size was determined by data saturation [28], which meant no further themes could be identified in the analysis [29]. When no new themes emerged during the interview with the 16th CHW, two additional CHWs were recruited for interviews to determine that data saturation was reached, so recruitment was halted after the 18th CHW was interviewed.

### Data collection

All interviewers were trained in qualitative data collection and experienced in qualitative interviewing. To improve inter-interviewer reliability, the research team developed a semi-structured interview guide and used it in the interviews.

The semi-structured interview outline was developed based on the study's research objectives and an extensive literature review. After a thorough discussion by the research team, interview outlines were designed for two different situations. The interview questions were piloted by interviewing two community health service workers before the formal interview and modified accordingly. All pilot interviewees were not included in the formal survey. The core content of the interview outline is shown in Table 1.

**Table 1:** The core content of the interview outline

**Situation type**	**Interview Outline**
Situation 1: Community health workers have conducted first aid training for older adults in the community	What kind of first aid knowledge and skills have you developed for older adults in the community; how did you conduct the first aid training? Were there any problems or obstacles encountered during the first aid training? What was the effectiveness of the first aid training for older adults in the community? What are your views on providing first aid training to older adults in the community?
Situation 2: Community health workers have not conducted first aid training for older adults in the community	Why don't you conduct first aid training for older adults in the community? What are your views on providing first aid training to older adults in the community?

The researchers conducted face-to-face, semi-structured in-depth interviews with the participants, allowing the researchers to obtain detailed descriptions to delve into the interviewees' perspectives. Interviews were conducted in the quiet community health center lounge. The purpose and main content of the interview were explained to the interviewees before the interview, and consent was obtained to record the interview. The duration of each interview was 20–30 min. During the interview, the researcher applied interview techniques, including questioning, repeating, following up, and responding to encourage them to express their true opinions and feelings. The interviews ended when the data were saturated, and no new themes emerged.

### Data analysis

The interviews were conducted in Chinese. After each interview, the written transcripts were reviewed jointly by the researcher and the CHWs to check the accuracy of statements and interpretations and to ensure the confirmability of data. In addition, two researchers (FZ, YW) transcribed the recorded interviews verbatim into Chinese within 24 h, and the transcribed transcripts were checked verbatim for accuracy by another researcher (GY).

This study used a content analysis approach. Two researchers (GY, CL) analyzed the data using Krippendorff's thematic clustering technique [30]. The researchers first read the interview transcripts repeatedly to obtain an understanding of the data as a whole; then coded the vital content in the transcript; similar and related codes were grouped to form categories and subcategories; finally, on the basis of the codes, certain ideas were categorized and conceptualized more systematically to form themes. Independent analysis of the data by two researchers was considered an important indicator of dependability [31]. The coding process was independently conducted by two researchers (GY, CL), followed by cross-checking of data by one researcher to improve the accuracy of data analysis (SL). Controversial themes were also discussed and negotiated by all authors, and the study results reflect an accurate interpretation of the data. In addition, the illustrative quotations used for the results were initially translated into English by two researchers (GY, CL) and then back-translated into Chinese by another researcher (SL) for comparison with the original text to ensure translation quality and consistency. The participants' views were recorded and transcribed precisely, which added to the study's credibility [32]. More specifically, the exact data that provided the basis for the analysis are provided in this study.

### Ethical considerations

This study was approved by the Academic Ethics Committee of Nanfang College of Guangzhou, Permit Number: NF2022040801. The study was conducted in strict accordance with the Declaration of Helsinki. Participants were informed of the purpose and voluntary of the study prior to conducting the survey, and all participants provided written informed consent.

## Results

### Participant characteristics

A total of 18 participants were included in the study. None of the potential participants interviewed declined to take part in the study. Seven CHWs were from urban community health service centers, and 11 were from rural community health service centers. The average age of the participants was 36.78 years. Specific participant characteristics are shown in Table 2.

**Table 2:** Characteristics of the participants (*N* = *18*)

**Coding**	**Age** **(years)**	**Gender**	**Level of education**	**Work time (years)**	**Community source**	**FA training experience for** **older adults** **(Y/N)**
P01	35	M	College	11	Rural	N
P02	40	M	Undergraduate	20	Rural	Y
P03	42	F	College	20	Rural	N
P04	35	M	College	10	Rural	Y
P05	24	F	College	1	Rural	N
P06	42	M	Undergraduate	19	Urban	Y
P07	45	F	Undergraduate	23	Rural	N
P08	24	F	College	1	Rural	N
P09	33	M	Undergraduate	11	Rural	N
P10	47	F	College	10	Rural	Y
P11	36	F	Undergraduate	11	Urban	N
P12	49	M	College	26	Urban	N
P13	27	F	Undergraduate	6	Urban	N
P14	26	F	Undergraduate	7	Rural	Y
P15	25	F	College	4	Urban	Y
P16	29	F	Undergraduate	5	Urban	N
P17	27	F	Undergraduate	4	Urban	N
P18	34	F	Undergraduate	5	Rural	Y

Abbreviations:* P* participant*, M male, F female, Y yes, N no*

### Themes

The perceived challenges and barriers to delivering first aid training to older adults were identified through interviews with CHWs as older adults-level barriers, community health workers-level barriers, management systems–level barriers, and society-level barriers. The specific themes and sub-themes are shown in Table 3.

**Table 3:** Themes and subthemes derived from the data

**Themes**	**Subthemes**
Older adults-level barriers	Limited physical functions
	Lack of motivation and interest
	Limited literacy and education
Community health worker- level barriers	Lack of first aid-related skills and knowledge
	Work overload
	Age discrimination
Management systems-level barriers	Lack of human resources allocation
	Lack of financial support
Social-level barriers	Decline in social integrity
	Lack of trust in community health workers

#### Theme I Older adults-level barriers

##### Limited physical functions

Aging can lead to a decline in the physical functions of older adults, such as diminished mobility, memory, and comprehension. The physical condition of older adults is a concern for CHWs and hinders the willingness of CHWs to provide FA training to older adults. Older adults' s memory, comprehension, and physical functions and conditions also have a negative impact on the effectiveness of FA training for them.

*"We have conducted FA education to older adults, but we found that the participation of older adults was poor, this kind of professional knowledge training was not attractive to them, and they cannot remember or understand the content of the training."* (P02).

*"Most older adults have limited mobility, poor comprehension, and some of them have deafness and tinnitus."* (P04).

##### Lack of motivation and interest

From the perspective of CHWs, older adults often underestimate the importance and urgency of learning FA. The lack of desire and motivation to engage in FA training among older adults is a serious problem.

*"We have conducted FA training for community residents in the community, and there are older adults who will participate in FA training, but not many… some older adults have to take care of their families and don't have time, and most older adults think they won't have an accidental injury."* (P06).

##### Limited literacy and education

Due to the varying literacy level of older adults, there are barriers to communication between CHWs and older adults, making FA education more challenging. On the other hand, CHWs, as professionals, may intentionally or unintentionally use medical terms when communicating with older adults. These medical terms are more difficult for older adults to understand, especially some older adults with low literacy levels. This also leads to communication difficulties and complicates FA training for older adults*.*

*" Older adults are generally not well educated; we popularize some first aid knowledge to them, but they do not understand, and I am helpless; I do not know how to make them understand.* (P14).

*"We have carried out FA skills training for older adults, and few older adults participated. Many of them participated in the training for small gifts and rarely actively participated in the training for their safety and health. Some rural older adults have low literacy and do not understand medical terminology."* (P18).

#### Theme II Community health worker- level barriers

##### Lack of first aid-related skills and knowledge

Another challenge to expanding FA education for older adults is that few CHWs have the necessary expertise or experience in FA training field. Inadequate FA education among CHWs makes them feel unqualified to provide FA training to older adults.

*"The CHWs are all inadequate in FA and have not undergone rigorous and systematic training in FA skills and are not equipped to conduct FA training."* (P02).

*"The current level of FA skills and knowledge of CHWs are both inadequate, and coupled with the lack of facilities, we are not up to the task of training older adults in FA."* (P07).

##### Work overload

Regarding the coronavirus outbreak, China has not slacked up on its surveillance or control. Working to prevent and manage epidemics and collecting nucleic acids are priorities for the community at large. The epidemic has consumed a lot of the workforce, materials, and resources. No more energy and focus on FA training for older adults. In addition, CHWs are concerned about the already overwhelmed workload within their current responsibilities and are reluctant to add additional tasks.

*"There are many tasks in community health work, coupled with the coronavirus epidemic, the workload is heavy, and now there is little rest time, and there is no extra time to organize FA training for older adults in the community.* (P11).

*"Due to the impact of the epidemic, most of our work was taken up by some tasks of epidemic prevention and control, so it was difficult to have extra energy and time to organize other activities."* (P13).

##### Age discrimination

Some community workers have age discrimination against older adults. CHWs may assume that older adults cannot acquire sophisticated FA skills because of negative generalizations about their literacy, physical function, and comprehension.

*"Older people who participate in FA training may not learn or apply it even if they learn it, or they may be even less likely to apply it to people around them for fear of causing disputes."* (P02).

*" FA knowledge is more boring for older adults, and they have little interest in learning. In addition, we are not sure about the ability of older adults to judge FA situations; CPR and other FA operations also have certain physical requirements and most of older adults have limited physical strength. Therefore, it is not certain whether FA education can be provided to them."* (P16).

#### Theme III Management systems-level barriers

##### Lack of human resources allocation

Due to the understanding and memory of older adults, FA training for older adults needs to be carried out continuously, and it is meaningless perform FA training for older adults only once. However, due to the lack of community health workforce, it is difficult for CHWs to carry out continuous FA training.

*"Lack of comprehensive quality training team, some CHWs are not familiar with teaching and training work, and lack of training experience."* (P05).

*"Without regular retraining and frequent drills, it is difficult for older adults to consolidate their FA skills and apply them proficiently in emergencies. However, CHWs also has a heavy workload, and our limited* workforce *makes it difficult to have the time and energy to provide continuous FA training to older adults."* (P12).

##### Lack of financial support

Conducting training activities involves financial issues. FA training requires resources, space, materials, and models, such as mannequins for CPR instruction. Furthermore, CHWs need to be professionally trained. All of these require financial support. However, there is insufficient economic policy support due to insufficient attention from the health administration.

*"FA training requires technical and financial support, and there are some financial difficulties in community health centers."* (P09).

#### Theme IV Social-level barriers

Fraudulent incidents among older adults are frequent, and the decline in social integrity has led to a heightened sense of caution within this age group. In addition, some older adults have severe doubts regarding the competency of healthcare workers at community health service centers because of their negative impressions of CHWs. This also leads to resistance or distrust of o*lder adults towards FA training conducted by CHWs.*

*"Some older adults distrust us because some private institutions used to provide free health education and training to older adults, but their main purpose was to sell health care equipment to older adults…So this has led to some older adults having a distrustful attitude toward health education activities now, which also makes it difficult to organize older adults to actively participate when we carry out activities again."* (P15).

"*Older people have more trust in hospital doctors, and they feel that the healthcare workers in community health service centers are not skilled, so when activities are conducted, they usually do not actively participate."* (P10).

## Discussion

Although the current research emphasizes the importance and necessity of FA knowledge and skills training for older adults [33], this is not universally accepted. It is worth noting that the results of this study indicate that CHWs perceive multifaceted barriers and challenges to conducting community-based geriatric FA training.

Ageism among older adults by CHWs is the most fundamental factor that prevents them from providing FA education to older adults. "Ageism" refers to attitudes and differential treatment of older adults by healthcare providers who make negative value judgments because of advanced age [34]. CHWs are concerned about the physical functioning of older adults and the retention of knowledge and skills they have learned in FA courses, and these inherent biases against older populations have been confirmed in other studies [35, 36]. Incompetence is the most common negative geriatric stereotypes of older adults [37]. This has also led to the exclusion of older adults from the target population of most FA training programs [38]. According to Prince et al. (2015), age should not dictate access to treatment and care [39]. Age is also not a justification for denying older adults access to education. Multiple studies have demonstrated that older adults are capable of learning and performing FA skills [40, 41]. Most older adults with self-care abilities have a strong desire to learn FA knowledge and skills so that they can save themselves or their families in times of crisis [42, 43]. Therefore, it is vital for CHWs to alter their stereotypical image of older adults, and they should also be aware of the significance and necessity of FA training for older adults.

Some CHWs are aware of the importance of FA training for older adults; nevertheless, personnel shortages and job pressure render them helpless. In fact, China has a far lower ratio of CHWs to residents than other developed countries [19]. One study found that some jobs are omitted when CHWs perceive resource adequacy and understaffing [44]. Unfortunately, the omitted tasks are not only found in programs for FA education for older adults. In China, community health services encompass disease prevention, health education guidance, health care, and medical treatment [45]. CHW staff shortages and heavy workloads are prevalent throughout the health field, which has led to community workers' services actually remaining far from their objectives [46]. Consequently, the same misses exist in activities such as the management of chronic diseases, screening for diseases, and health education carried out by CHWs [44]. On the other hand, Taylor et al. (2021) contend that when CHWs are seen as bearers of more and more roles and tasks, this also means that they are focused on current work tasks without proactively identifying new needs [47]. Additionally, their broad responsibilities and lack of expertise leave CHWs ambiguous about their job roles and make them feel overwhelmed and powerless [48]. Workload overload, insufficient first aid knowledge and skills, and lack of professional support limited CHWs' willingness to deliver FA training to older adults in the present study. A systematic review showed that the provision of professional training and related service skills can motivate CHWs to provide related services [21]. Therefore, their roles and FA knowledge and skills can be clarified and enhanced by providing organized training, which will also promote the motivation of CHWs to conduct FA education for older adults.

However, recent research indicates that simply educating and training CHWs on FA knowledge and competences is insufficient. In fact, training in instructional techniques and instructional design is even more critical. The current knowledge and content of FA training typically assume that learners have advanced literacy skills as well as cultural and cognitive strengths. The majority of FA courses are meant to be more suitable for highly educated individuals. A scientific investigation corroborate this assertion [49]. Both text-based learning and medical terminology represent substantial problems for older adults in FA instruction. According to the findings of this study, older adults did not comprehend a portion of medical terminology and the content of professional FA knowledge. Excessive medical terminology is also the most significant factor in communication barriers between healthcare workers and older adults [50]. Perhaps, FA courses should be tailored and adapted for the elderly population. Specific aspects such as age, education level, and physical function need to be considered to suit the needs of the geriatric population. As revealed in a study by Dolenc et al. (2021), the complexity of FA procedures was a major barrier to older people's participation in FA training; older adults prefer shorter and more focused FA training sessions due to their weakened psycho-physical state and limited capacity for concentration [51]. Further evidence from Canada demonstrates that older adults will be more receptive if health information is readily accessible and understandable [52].

Health system and policy support may be both a barrier and a facilitator for CHWs to carry out their work, according to another significant finding of this study. Ambiguous policies and perspectives may hinder CHWs from implementing health intervention programs, while official government and health system support facilitates implementation [45]. Unfortunately, there is currently no national program for FA training for the elderly population in China [53]. In fact, Dolenc et al. (2022) surveyed 54 European countries and also did not find any FA training programs for older adults [42, 43]. FA training for older adults has not received much attention from health systems and governments.

On the other hand, conducting a community-based first aid skills training program requires staff, financial, facility, and space support. However, limited by the local economy and government interests, not all CHWs have access to the necessary support, and funding is severely lacking in difficult situations [21]. Because of this, community health centers lack the latest medical technology, equipment, medical resources, and lack of professional and qualified medical staff, which complicates the "siphon effect" where the majority of Chinese patients prefer to go to large hospitals rather than community hospitals [54]. Additionally, CHWs have a low degree of education, professionalism, and medical knowledge and thus have a poor reputation [45]. For these reasons, it is difficult for CHWs to earn the trust of older adults. CHWs also struggle to effectively execute training activities [55]. On the other hand, influenced by the unstable social circumstances, the visibility of CHWs in the community also affects the trust of community residents in them [21]. Therefore, under the existing conditions, the training of CHWs and investment in equipment should be increased to promote the overall quality of the community health service team in order to fundamentally establish a positive image of CHWs, with the ultimate goal of establishing their credibility and fostering trust between them and older adults.

## Limitations and recommendations

This study has explored barriers and challenges to FA training for older adults only from the perspective of CHWs, and further exploration from the perspective of older adults is necessary. In addition, future research could investigate the differences in value between FA training for older and younger persons from the perspective of community health workers, which was not investigated in this study. Since the sampling sites were selected based on the researchers' local community, there may be some selection bias. Therefore, future research could improve generalizability by considering surveys in other areas. Although there are some limitations, the study provides some insight into the current barriers for CHWs to provide FA training to older adults.

## Conclusion

The geriatric population has received little attention in FA education. The findings of this study provide a new perspective on the barriers and challenges faced in providing FA training to older adults by CHWs. The findings also provide insight into the development of FA education for older adults. The age discrimination of CHWs toward older adults should be primary considerations when designing and implementing community-based FA education programs for older adults. Secondly, CHWs must strive to elevate their professional standards, establish a positive reputation, and build trusting relationships with older adults. Importantly, FA training for older adults in the community requires policies and government funding.

## Data Availability

The datasets used and analysed during the current study can be obtained from the corresponding author on reasonable request.

## Abbreviations

CHW/CHWs: Community Health Worker / Community Health Workers
FA: First Aid
CPR: Cardiopulmonary Resuscitation
